# The pathological role of advanced glycation end products-downregulated heat shock protein 60 in islet β-cell hypertrophy and dysfunction

**DOI:** 10.18632/oncotarget.8604

**Published:** 2016-04-05

**Authors:** Siao-Syun Guan, Meei-Ling Sheu, Rong-Sen Yang, Ding-Cheng Chan, Cheng-Tien Wu, Ting-Hua Yang, Chih-Kang Chiang, Shing-Hwa Liu

**Affiliations:** ^1^ Institute of Toxicology, College of Medicine, National Taiwan University, Taipei, Taiwan; ^2^ Institute of Nuclear Energy Research, Atomic Energy Council, Executive Yuan, Taoyuan, Taiwan; ^3^ Biomedical Sciences, College of Life Sciences, National Chung Hsing University, Taichung, Taiwan; ^4^ Department of Orthopaedics, College of Medicine, National Taiwan University, Taipei, Taiwan; ^5^ Department of Geriatrics and Gerontology, College of Medicine, National Taiwan University, Taipei, Taiwan; ^6^ Department of Otolaryngology, National Taiwan University Hospital, Taipei, Taiwan; ^7^ Department of Integrated Diagnostics and Therapeutics and Internal Medicine, College of Medicine and Hospital, National Taiwan University, Taipei, Taiwan; ^8^ Department of Medical Research, China Medical University Hospital, China Medical University, Taichung, Taiwan; ^9^ Department of Pediatrics, National Taiwan University Hospital, Taipei, Taiwan

**Keywords:** diabetes, advanced glycation end products, β-cell hypertrophy, heat shock protein 60, Pathology Section

## Abstract

Heat shock protein 60 (HSP60) is a mitochondrial chaperone. Advanced glycation end products (AGEs) have been shown to interfere with the β-cell function. We hypothesized that AGEs induced β-cell hypertrophy and dysfunction through a HSP60 dysregulation pathway during the stage of islet/β-cell hypertrophy of type-2-diabetes. We investigated the role of HSP60 in AGEs-induced β-cell hypertrophy and dysfunction using the models of diabetic mice and cultured β-cells. Hypertrophy, increased levels of p27^Kip1^, AGEs, and receptor for AGEs (RAGE), and decreased levels of HSP60, insulin, and ATP content were obviously observed in pancreatic islets of 12-week-old *db/db* diabetic mice. Low-concentration AGEs significantly induced the cell hypertrophy, increased the p27^Kip1^ expression, and decreased the HSP60 expression, insulin secretion, and ATP content in cultured β-cells, which could be reversed by RAGE neutralizing antibody. HSP60 overexpression significantly reversed AGEs-induced hypertrophy, dysfunction, and ATP reduction in β-cells. Oxidative stress was also involved in the AGEs-decreased HSP60 expression in β-cells. Pancreatic sections from diabetic patient showed islet hypertrophy, increased AGEs level, and decreased HSP60 level as compared with normal subject. These findings highlight a novel mechanism by which a HSP60-correlated signaling pathway contributes to the AGEs-RAGE axis-induced β-cell hypertrophy and dysfunction under diabetic hyperglycemia.

## INTRODUCTION

The onset of insulin resistance of the peripheral tissues triggers hyperinsulinemia coupled with increased β-cell mass and hypertrophy of existing β-cells, which in turn leads to gradual β-cell exhaustion and dysfunction and eventually β-cell mass loss by apoptosis [1-4]. Weir and Bonner-Weir have reviewed that during the stage of compensation in the progression of diabetes (insulin resistance/obesity), the increase in insulin secretion due to an increase in β-cell mass, which has been shown in autopsy studies in humans and several rodent models [5]. The increased β-cell number (hyperplasia) and β-cell hypertrophy probably contribute to the increased β-cell mass during β-cell compensation. Butler *et al*. have also found that relative β-cell volume is increased in obese nondiabetic humans, in parallel to hyperinsulinism, *via* an increased neogenesis mechanism; obese with type-2 diabetes (T2D) *versus* nondiabetic obese have a 63% deficit in relative β-cell volume [6]. Cho *et al.* have observed the increased β-cell size (approximately 30% larger) and the increased ratio of cytoplasm per nucleus area in type 2 diabetic patients compared with normal subjects [7]. However, the mechanism of increased β-cell mass or hypertrophy during early stage of T2D still remains to be clarified.

Advanced glycation end products (AGEs) are produced from non-enzymatic reactions between reducing sugars and amino groups of proteins. Increasing evidence shows that the accumulation of AGEs conducts the characteristic features in diabetes [8]. AGEs may exert their biological effects by altering protein function, causing abnormal interactions among matrix proteins, and interfering with cellular functions through the receptor for AGEs (RAGE) [9]. The interaction of AGEs with RAGE triggers an intracellular signaling transduction and activates the transcription factor NF-κB, leading to chronic inflammation and consequent cellular and tissue impairment [10]. AGEs have been demonstrated to contribute to β-cell apoptosis and dysfunction, leading to the decrease in the insulin synthesis and secretion [11, 12]. In addition, AGEs have been shown to interfere with the β-cell function *via* impairing mitochondrial function [13]. Under diabetic condition, AGEs-induced cell hypertrophy was observed in various cells, including renal tubular cell, podocyte, glomerular mesangial cell, cardiomyocyte [14-17]. However, the regulatory role of AGEs on β-cell hypertrophy remains to be clarified.

Mitochondrial heat shock protein 60 (HSP60) is a specific molecular chaperone and an important protein for the maintenance of mitochondrial integrity and cell viability [18, 19]. HSP60 works together with its co-chaperone HSP10 to assist proper folding and assembly of mitochondrial proteins in response to oxidative stress [19, 20]. HSP60 is crucial for the survival of cells under stress conditions, and *Hsp60* deficiency results in cellular apoptosis and early embryonic lethality in mice [21]. Mutations in the nuclear gene that encodes mitochondrial HSP60 in human (*HSPD1* gene) are associated with two neurodegenerative diseases, hereditary spastic paraplegia and MitChap60 disease [22, 23]. It has been shown that the expression of HSP60 was reduced in the hypothalamus of type 2 diabetic patients and mice [24]. Both mouse hypothalamic cells with knockdown of *Hsp60* and mice with heterozygous deletion of *Hsp60* exhibit mitochondrial dysfunction and hypothalamic insulin resistance [24], indicating that HSP60 may contribute to the regulation of mitochondrial function and insulin sensitivity in the hypothalamus under T2D condition. However, the role of HSP60 in the β-cell hypertrophy and dysfunction under diabetic condition is still unclear.

In this study, we hypothesize that AGEs induce β-cell hypertrophy and dysfunction through a HSP60 dysregulation pathway during the stage of islet/β-cell hypertrophy of T2D. We investigated the hypertrophy of islets/β-cells and the expressions of AGEs/RAGE and HSP60 and the role of HSP60 in the effects of AGEs on β-cell hypertrophy and dysfunction *in vitro* and *in vivo*. The islet hypertrophy and the expressions of AGEs and HSP60 in human pancreatic samples of diabetic patient were also examined.

## RESULTS

### Hypertrophy and expressions of AGEs, RAGE, and HSP60 in islets of diabetic mice

**Figure 1:**
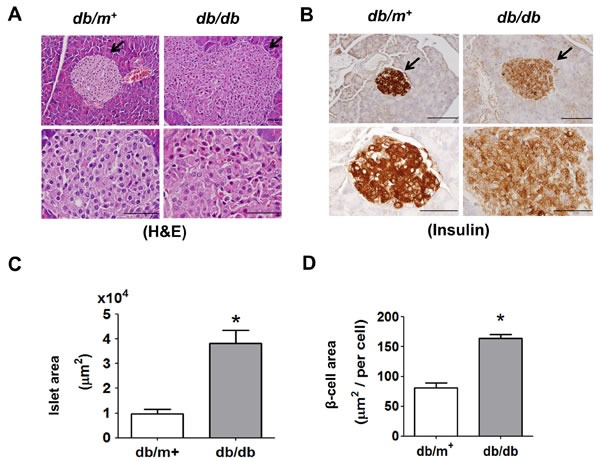
Histology and immunohistochemical staining for insulin in pancreatic islets of db/db diabetic mice Hematoxylin and eosin staining **A.** and immunohistochemical staining for insulin **B.** in pancreatic sections from *db/db* and *db/m*^+^ mice were shown. Original magnification, ×400, scale bar: 100 μm; x1000, scale bar: 50 μm. The arrows showed that indicated areas have enlarged scales. Moreover, the islet area **C.** and β-cell area **D.** in islets of *db/db* and *db/m*^+^ mice with 6 random areas per section was determined by ImageJ software. Data are presented as mean ± SEM. *P < 0.05, *db/db versus db/m*^+^ mice.

The body weight (38.38 ± 0.96 *versus* 25.24 ± 1.32 g, *n* = 10, *p* < 0.05), fasting plasma glucose (354.2 ± 50.54 *versus* 101.1 ± 21.74 mg/dl, *n* = 10, *p* < 0.05), and serum insulin (6.86 ± 3.13 *versus* 1.10 ± 0.37 μg/l, *n* = 10, *p* < 0.05) in *db/db* mice were significantly increased as compared with *db/m^+^* mice. The stainings of H&E and insulin showed that islets were significantly displayed hypertrophy in *db/db* mice compared to *db/m^+^* mice (Figure 1A and 1B). The intensity of staining for insulin in islets of *db/db* mice was weaker than that of *db/m^+^* mice (Figure 1B). The islet area (Figure 1C) and β-cell area (Figure 1D) in islets of *db/db* mice was also significantly increased as compared with *db/m^+^* mice.

**Figure 2:**
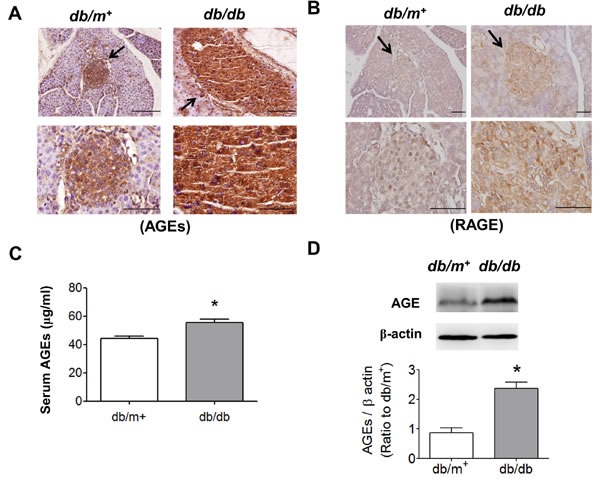
Immunohistochemical staining for AGEs and RAGE in pancreatic islets of db/db diabetic mice Immunohistochemical staining for AGEs **A.** and RAGE **B.** in pancreatic sections from *db/db* and *db/m*^+^ mice were shown. Original magnification, ×400, scale bar: 100 μm; x1000, scale bar: 50 μm. The arrows showed that indicated areas have enlarged scales. **C.** Serum AGEs detection was determined by ELISA. **D.** The protein expression of AGE-BSA in islets of *db/db* and *db/m*^+^ mice determined by Western blotting. Data are presented as means ± SEM (*n* = 10). *P < 0.05, *versus db/m*^+^ mice.

We further detected the expressions of AGEs and RAGE in pancreatic islet areas of *db/db* and *db/m^+^* mice by immunohistochemical staining. The result revealed that the expressions of AGEs (Figure 2A) and RAGE (Figure 2B) in pancreatic islets were prominently increased in *db/db* mice compared to *db/m^+^* mice. Moreover, the serum AGEs levels of *db/db* mice were markedly higher than *db/m^+^* mice (Figure 2C). The protein expression of AGE-bovine serum albumin (AGE-BSA) was also significantly increased in islets from *db/db* mice (Figure 2D).

**Figure 3:**
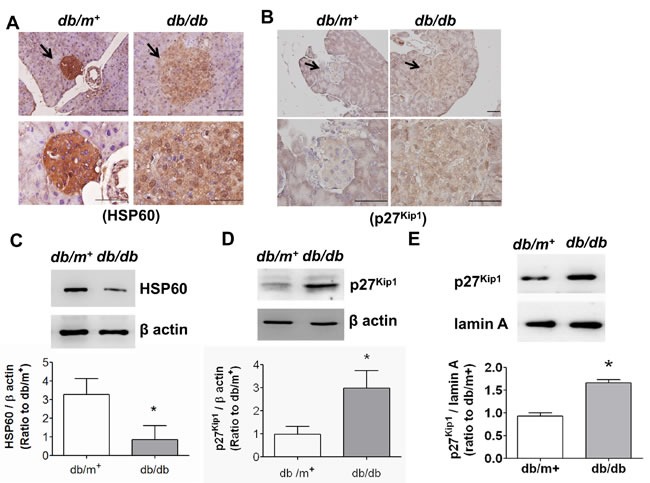
The expressions of HSP60 and p27Kip1 in pancreatic islets of db/db diabetic mice Immunohistochemical stainings for HSP60 **A.** and p27^Kip1^ **B.** in pancreatic sections from *db/db* and *db/m*^+^ mice were shown. Original magnification, ×400, scale bar: 100 μm; x1000, scale bar: 50 μm. The protein expressions of HSP60 **C.** and p27^Kip1^ **D.** in pancreatic islets isolated from *db/db* and *db/m*^+^ were determined by Western blotting. Moreover, the nuclear p27^Kip1^ protein expression was also determined **E.**. Protein levels were quantified by densitometry and normalized by β-actin levels. Data are presented as means ± SEM (*n* = 4). *P < 0.05, *versus db/m*^+^ mice.

We next tested the expressions of HSP60 and p27^Kip1^ (cell cycle arrest marker) in pancreatic islet areas of *db/db* and *db/m^+^* mice by immunohistochemical staining and immunoblotting. As shown in Figure 3, the HSP60 level was markedly decreased (Figure 3A) and the p27^Kip1^ level was obviously increased (Figure 3B) in islet areas of *db/db* mice compared to *db/m^+^* mice. Moreover, the decreased HSP60 protein expression (Figure 3C) and the increased p27^Kip1^ protein expression (Figure 3D) were also observed in pancreatic islets isolated from *db/db* mice. The p27^Kip1^ protein expression in the nuclear fraction of islets from *db/db* mice was also significantly increased (Figure 3E).

### AGEs induced HSP60 down-regulation and cell hypertrophy and decreased insulin and ATP contents in a rat β-cell line

**Figure 4:**
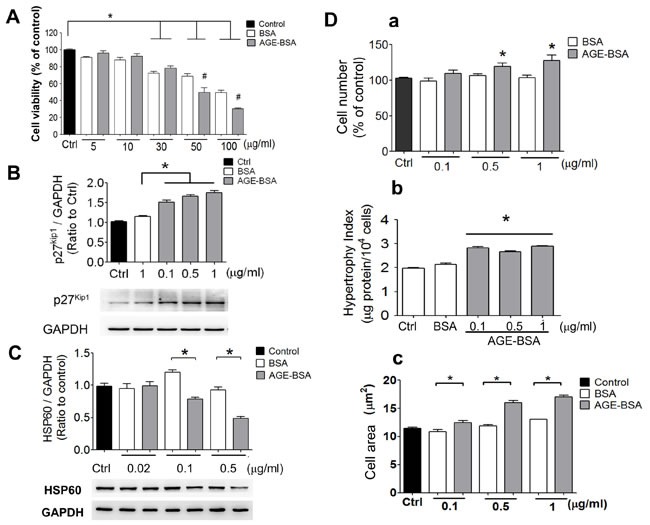
AGEs induce cell hypertrophy and decrease HSP60 expression in cultured β-cells Effects of AGEs on the cell viability **A.**, p27^Kip1^ protein expression **B.**, and HSP60 protein expression **C.** in RINm5f cells were shown. Cells were treated with AGE-BSA (5-100 μg/ml in A or 0.02-1 μg/ml in B and C) for 24 hours. Cell viability was determined by WST-8 assay. The protein expression was determined by Western blotting. Protein levels were quantified by densitometry and normalized by GAPDH levels. Moreover, effects of AGEs on the cell number (D-a), cell hypertrophy index (D-b), and cell area (D-c) of RINm5f cells were investigated. Cells were treated with AGE-BSA (0.1-1 μg/ml) for 24 hours. The viable cell number was determined by trypan blue exclusion assay. The cell hypertrophy index and cell diameter were measured as described under “Materials and Methods”. Data are presented as means ± SEM (n ≥ 5). *P < 0.05, *versus* BSA.

**Figure 5:**
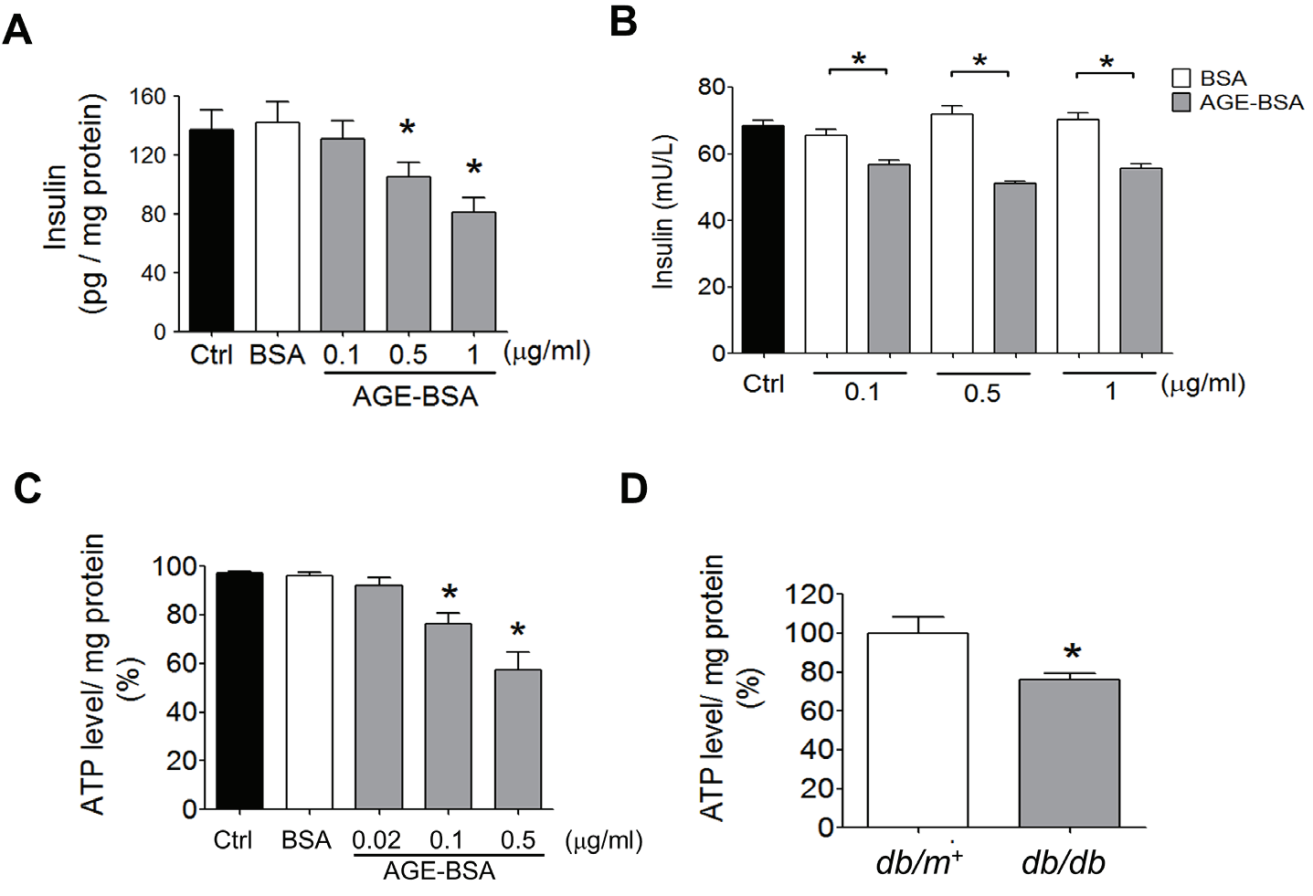
Effects of AGEs on insulin content and ATP production in β-cells RINm5f cells were treated with AGE-BSA and non-glycated BSA (0.02-1 μg/ml) for 24 hours. The cellular insulin content **A.** and insulin secretion in medium **B.** and cellular ATP content **C.** were measured. Data are presented as means ± SEM (n ≥ 5). *P < 0.05, *versus* BSA, ^#^*P* < 0.05, *versus* pM51/AGE-BSA. In some experiments, the ATP levels in islets of *db/db* and *db/m+* mice were detected **D.**. Data are presented as means ± SEM (n ≥ 10). *P < 0.05, *versus db/m+* mice.

AGE-BSA was no significant cytotoxicity in RINm5f cells at the concentrations of 5 and 10 μg/ml compared to control (Figure 4A). The p27^Kip1^ protein expression was markedly increased (Figure 4B) and the HSP60 protein expression was obviously decreased (Figure 4C) in low-concentration AGE-BSA (0.1-1 μg/ml)-treated RINm5f cells compared to BSA-treated group. Moreover, we further investigated the effects of low-concentration AGE-BSA on the cell number and hypertrophy. As shown in Figure 4D, the cell number, hypertrophy index, and cell area were increased by AGE-BSA at the concentrations of 0.1-1 μg/ml compared to BSA-treated group. On the other hand, the intracellular insulin levels and insulin secretion were significantly decreased by low-concentration AGE-BSA (0.1-1 μg/ml) (Figure 5A and 5B). Moreover, the intracellular ATP levels were significantly decreased in AGE-BSA-treated RINm5f cells (Figure 5C). In addition, the ATP levels in islets were also decreased in *db/db* diabetic mice compared to *db/m+* mice (Figure 5D).

### Involvement of AGEs-RAGE axis in cell hypertrophy and impairment of ATP production and insulin secretion in a rat β-cell line

**Figure 6:**
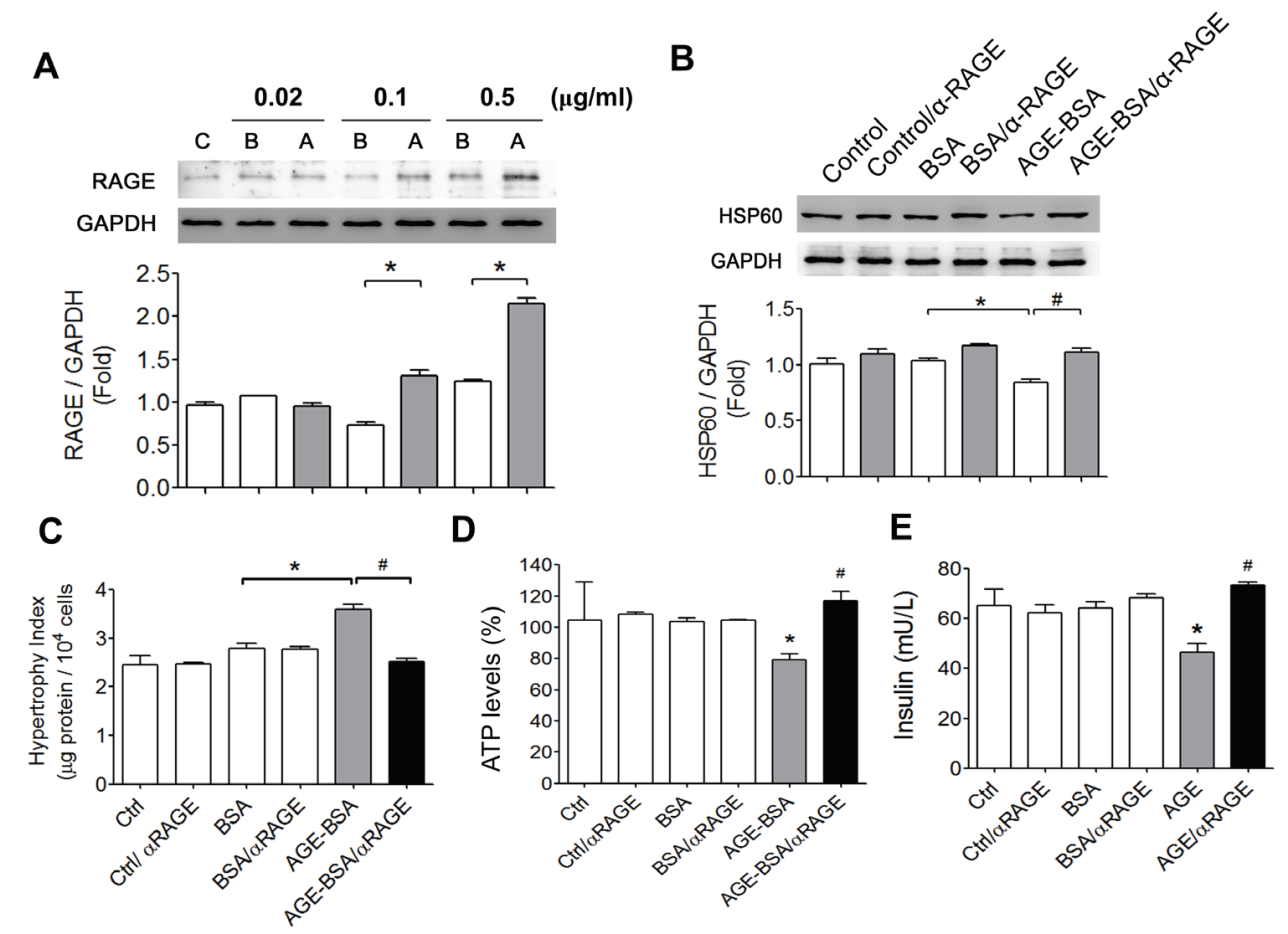
Involvement of AGEs-RAGE axis in the alterations of HSP60 protein expression, cell hypertrophy, ATP production, and insulin secretion in AGEs-treated β-cells **A.** The effect of AGEs on RAGE protein expression in RINm5f cells. Cells were treated with AGE-BSA or non-glycated BSA (0.02-0.5 μg/ml) for 24 hours. The protein expression was determined by Western blotting. Protein levels were quantified by densitometry and normalized by GAPDH levels. Data are presented as means ± SEM (n ≥ 5). *P < 0.05, *versus* BSA. C: control, B: BSA, A: AGE-BSA. (B-E) After the pretreatment of RAGE neutralizing antibody (10 μg/ml) for 1 hour, RINm5f cells were treated with AGE-BSA or non-glycated BSA (0.5 μg/ml) for 24 hours. The protein expression of HSP60 **B.**, cell hypertrophy index **C.**, ATP content **D.**, and insulin production **E.** were detected as described under “Materials and Methods”. Data are presented as means ± SEM (*n* = 4). *P < 0.05, *versus* BSA, ^#^*P* < 0.05, *versus* AGE-BSA.

We next investigated whether AGEs-RAGE axis was involved in the cell hypertrophy and the alterations in HSP60 expression, ATP production, and insulin secretion in cultured RINm5f cells. The protein expression of RAGE was significantly increased by low-concentration AGE-BSA (0.1 and 0.5 μg/ml) in RINm5f cells (Figure 6A). The decreased HSP60 protein expression in AGE-BSA-treated RINm5f cells was significantly reversed by neutralizing RAGE antibody treatment (Figure 6B). Moreover, the increased cell hypertrophy (Figure 6C) and the decreased ATP production (Figure 6D) and the decreased insulin secretion (Figure 6E) in AGE-BSA-treated RINm5f cells were also reversed by neutralizing RAGE antibody treatment.

### Overexpressed HSP60 inhibited cell hypertrophy and impairment of insulin secretion and ATP production in AGE-BSA-treated rat β-cell line

**Figure 7:**
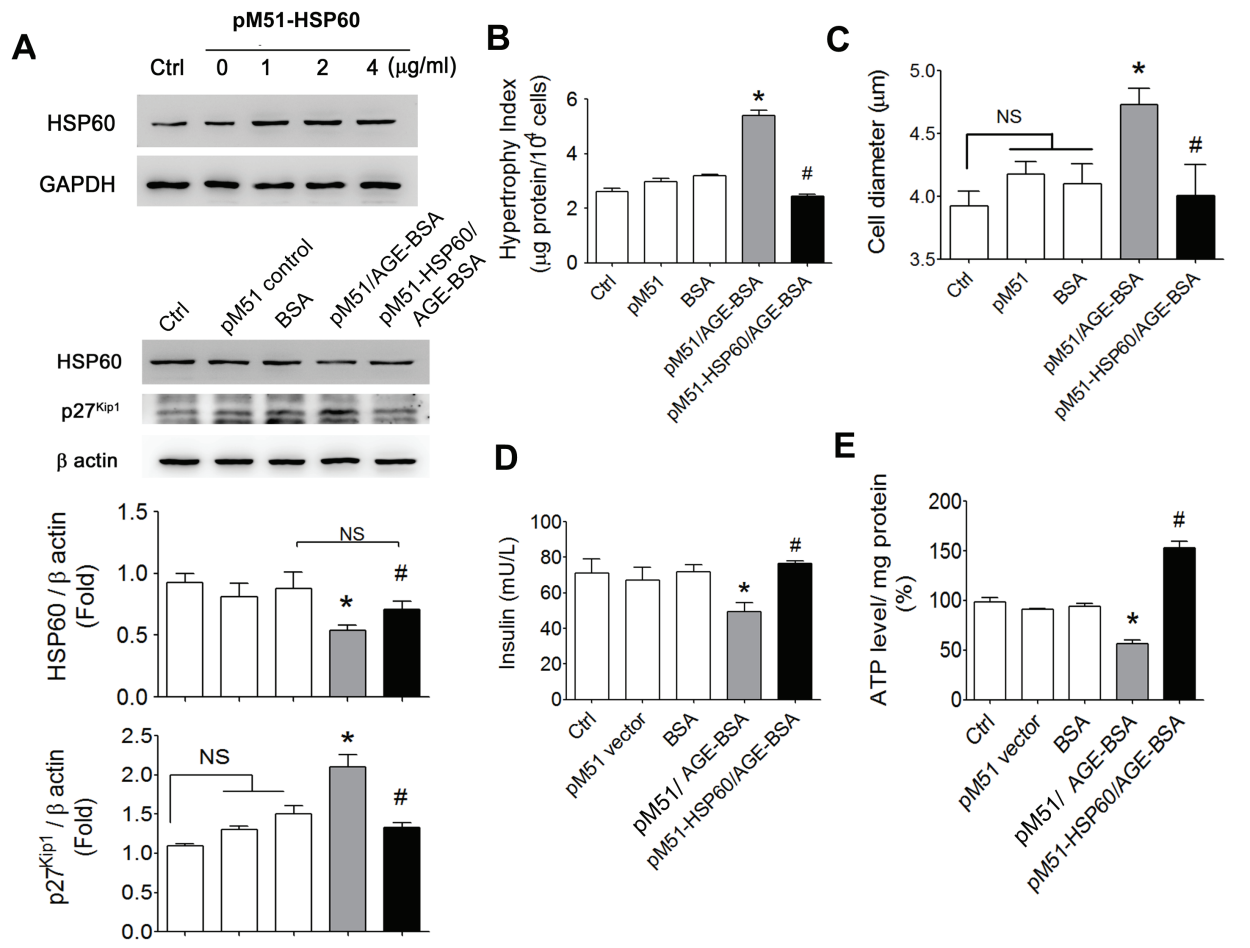
Effects of overexpressed HSP60 on cell hypertrophy and insulin secretion and ATP production in AGEs-treated β-cells RINm5f cells were transfected with pM51-HSP60 (0-4 μg/ml) for 48 hours. The pM51 empty vector was as a negative control. Transfection of pM51-HSP60 or pM51 vector control (1 μg/ml) for 48 hours, and then cells were treated with AGE-BSA and non-glycated BSA (0.5 μg/ml) for 24 hours. The protein expressions of HSP60 and p27^Kip1^ **A.**, cell hypertrophy index **B.**, cell diameter **C.**, insulin secretion **D.**, and ATP production **E.** were detected as described under “Materials and Methods”. Data are presented as means ± SEM (*n* ≥ 5). *P < 0.01, *versus* BSA, ^#^*P* < 0.01, *versus* pM51/AGE-BSA. NS: non-significant.

In order to clarify the role of HSP60 in AGEs-induced effects, the HSP60 was overexpressed in RINm5f cells. The protein levels of HSP60 were markedly increased in pM51-HSP60-transfected RINm5f cells. The decreased HSP60 protein expression and the increased p27^Kip1^ protein expression in AGE-BSA-treated RINm5f cells were significantly reversed by the overexpression of HSP60 compared to pM51 control vector transfection (Figure 7A). In addition, the results of hypertrophy index and cell diameter measurement revealed that AGE-BSA-increased cell volume was also significantly inhibited in pM51-HSP60-transfected cell (Figure 7B and 7C). Overexpression of HSP60 could also significantly reverse the decreased insulin secretion (Figure 7D) and ATP production (Figure 7E) in AGE-BSA-treated RINm5f cells.

### AGEs induced ROS production *via* RAGE in a rat β-cell line

**Figure 8:**
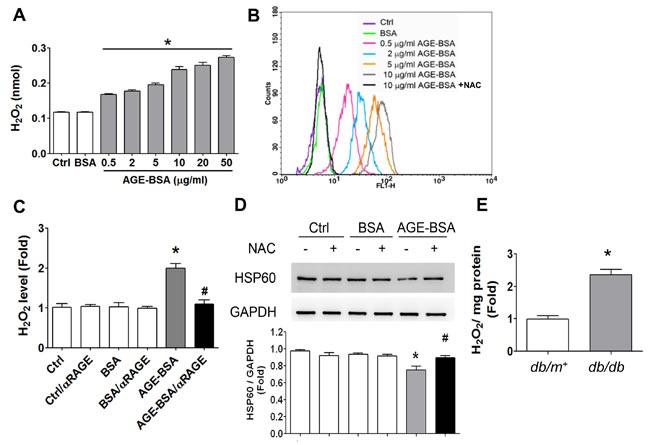
Oxidative stress is involved in the AGEs-RAGE axis-induced inhibition of HSP60 expression in β-cells RINm5f cells were treated with AGE-BSA (0.5-50 μg/ml) and non-glycated BSA (50 μg/ml) for 24 hours. **A.** The levels of cellular H_2_O_2_ were detected by ELISA. Data are presented as means ± SEM (*n* ≥ 5). *P < 0.05, *versus* BSA. **B.** ROS production was also determined by flow cytometric assay. NAC, N-acetyl-L-cysteine. **C.** RINm5f cells were treated with AGE-BSA and non-glycated BSA (10 μg/ml) for 24 hours in the presence or absence of RAGE neutralizing antibody. The levels of cellular H_2_O_2_ were detected by ELISA. **D.** Effect of antioxidant N-acetyl-L-cysteine (NAC) on HSP60 protein expression in AGE-BSA-treated RINm5f cells. After pretreatment with NAC (2 mM) for 1 hour, the cells were treated with AGE-BSA or non-glycated BSA (0.5 μg/ml) for 24 hours. The protein expression of HSP60 was determined by Western blotting. Protein levels were quantified by densitometry and normalized by GAPDH levels. Data are presented as means ± SEM (*n* = 4). *P < 0.05, *versus* BSA, ^#^*P* < 0.05, *versus* AGE-BSA. In some experiments, the H_2_O_2_ productions in islets of *db/db* and *db/m+* mice were measured **E.**. Data are presented as means ± SEM (*n* ≥ 10). **P < 0.01, *versus db/m+* mice.

AGE-BSA (0.5-50 μg/ml) increased ROS production in RINm5f cells in a dose-dependent manner (Figure 8A and 8B). The increased ROS production in AGE-BSA-treated RINm5f cells could be significantly reversed by both antioxidant N-acetyl-L-cysteine (Figure 8B) and neutralizing RAGE antibody (Figure 8C). Moreover, N-acetyl-L-cysteine could also significantly reverse the decreased HSP60 protein expression in AGE-BSA-treated RINm5f cells (Figure 8D). In addition, the ROS production was also increased in islets of *db/db* diabetic mice compared to *db/m^+^* mice (Figure 8E).

### Hypertrophy and AGEs and HSP60 expressions in pancreatic islets of diabetic patient

**Figure 9:**
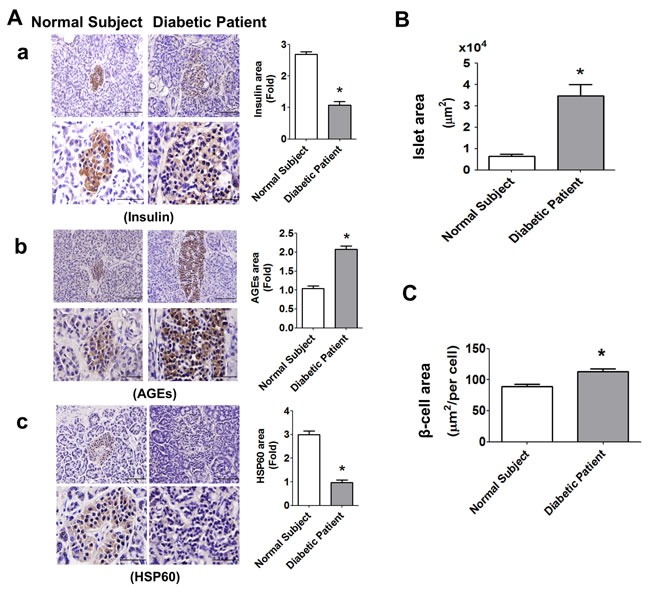
Immunohistochemical staining for insulin, AGEs, and HSP60, in the pancreatic islets of normal subject and diabetic patient The immunohistochemical staining for insulin (A-a), AGEs (A-b), and HSP60 (A-c) were performed on the pancreatic sections (islet areas) of normal subject and diabetic patient. Original magnification, ×400, scale bar: 100 μm; x1000, scale bar: 50 μm. Moreover, the islet area **B.** and β-cell area **C.** in islets of normal subject and diabetic patient with 6 random areas per section was determined by ImageJ software. Data are presented as mean ± SEM. *P < 0.05, diabetic patient *versus* normal subject.

The pancreatic islets (insulin-positive staining) in elderly type-2 diabetic patient (Figure 9A) showed hypertrophy as compared with normal elderly subject. The intensity of staining for insulin in islets of diabetic patient was weaker than that of normal subject (Figure 9A-a). The results of immunohistochemical staining also showed that the intensity of AGEs staining was significantly increased (Figure 9A-b), but the intensity of HSP60 staining was significantly decreased (Figure 9A-c) in islets of diabetic patient. In addition, the islet area (Figure 9B) and β-cell area (Figure 9C) in islet of diabetic patient was significantly increased as compared with normal subject.

## DISCUSSION

Pancreatic β-cell mass is determined by a dynamic balance between the rates of β-cell growth (hyperplasia, hypertrophy) and β-cell death (apoptosis) [25]. However, several studies have showed that hypertrophied β-cell is less prone to apoptosis in gene knockout mice [26] and glucose/insulin infusion rats [27]. Crawford *et al*. indicated that the *CTGF* heterozygous (*CTGF*^−/−^) animals showed increased β-cell size and p27^Kip1^ levels, but there were no β-cell apoptosis in WT or *CTGF* mutant animals [26]. Similarly, in a hyperglycemic-hyperinsulinemic animal model, the β-cell mass was significantly increased about 70% by increasing 30% individual β-cell size and 400% neogenesis and replication activation [27]. Nevertheless, immunohistochemical staining of β-cells in high glucose and high insulin rats represented that the apoptosis rate was very low, and no difference could be observed among the experimental groups [27]. Previous reports showed that high fat diet intake [28] and β-cell-specific Cdkal1 knockout mice [29] induced β-cell hypertrophy and increased β-cell/islet area and β-cell mass content in the pancreas due to chronic hyperglycemia state. In addition, chronic hyperglycemia induced by 85-95% pancreatectomy exhibited an onset of β-cell hypertrophy in rats [30]. The process of AGEs formation is particularly enhanced in diabetes, which contributes the mechanism in the pathogenesis of diabetic complications [31]. In T2D patients, the serum AGEs levels of diabetic patients were raised about 10 years after onset [32]. The high levels of serum AGEs was also observed in streptozotocin-induced hyperglycemic rats [33]. Several studies have shown that high-concentration AGEs (> 50 μg/ml) induced cytotoxicity, which may cause diabetic complications, including cardiovascular disease [34], nephropathy [35], and retinopathy [36]. A recent study indicated that AGEs contributed to the development of neurodegenerative diseases [37]. Byun *et al.* indicated that AGEs at the concentration of 20 μg/ml could promote human microglial cell apoptosis for neurodegenerative disorders development [38]. In the present study, we observed that AGEs levels in pancreatic islets of 12-week-old *db/db* mice were significantly increased. The size of pancreatic islet/β-cells was also significantly enhanced in *db/db* mice. Moreover, the low-concentration AGEs (0.1-5 μg/ml) significantly caused β-cell hypertrophy with no apoptosis *in vitro*. Therefore, we consider that insulin-producing cells may be more susceptible to AGEs toxicity than other cells. These results indicate that AGEs are involved in the islet/β-cell hypertrophy during diabetic condition. On the other hand, the accumulating evidence indicated that RAGE, a signal transduction receptor for AGEs, was involved in the pathogenesis of diabetes and its complications [39]. RAGE has been shown to participate in the apoptosis evoked by AGEs in β-cells [14]. In this study, we also observed that RAGE protein expression was increased in the islets of diabetic mice and AGEs-treated β-cell line. RAGE neutralizing antibody could significantly reverse the AGEs-induced cell hypertrophy, abnormality of ATP production, and insulin secretion impairment in cultured β-cell line. Therefore, these results suggest that AGEs-RAGE axis may be involved in the β-cell hypertrophy and dysfunction.

In several insulin resistant and diabetic rodent models, islet β-cell hypertrophy and hyperplasia occur to increase β-cell mass during β-cell compensation stage in response to insulin resistance and hyperglycemia [40]. It has been shown that oxidative stress-induced opening mitochondrial permeability transition pore (MPTP) causes ATP production breakdown, which is involved in the cardiac hypertrophy [41]. In addition, recent evidence showed that functional impairment of β-cell mitochondria is a major contributor for insulin secretory defects in T2D patients [42]. Although insulin secretion is stimulated by a number of stimulants, the oxidative mitochondrial metabolism is a central response for glucose-induced insulin secretion in human islets [43]. An impairment of oxidative mitochondrial metabolism may interfere with the ability of K_ATP_ channels closure and impair the β-cell electrical activity and insulin secretory function *via* the reduced mitochondrial ATP production. AGEs have been shown to increase the cytosolic ROS, which causes mitochondrial dysfunction in cardiomyocytes [44]. Melinda *et al.* also indicated that AGEs directly decreased the β-cell function, resulting from the decreased ATP production and manganese superoxide dismutase activity in mitochondria [18]. In the present study, we observed that low-concentration AGEs reduced ATP production and insulin secretion in cultured β-cells and induced β-cell hypertrophy. Interestingly, such a low dosage of AGEs is enough to cause ATP production down-regulation and decrease glucose-stimulated insulin secretory function. Therefore, we suppose that the accumulation of slight AGEs at β-cells may enhance the progress in development of early T2D.

A study has shown that the GroESL chaperone, composed of GroES and GroEL subunits (the bacterial Hsp10 and *Hsp60*), assists nascent polypeptides to reach a native conformation [45]. Two human inherited diseases of the nervous system, spastic paraplegia (SPG13) and MitCHAP60 disease, showed the mutations in Hsp60, which contributed to mitochondrial dysfunction [22, 23]. A recent study in HSP60 deficiency mice showed that HSP60 haplo-insufficiency is sufficient to cause a late onset motor neuron disorder [46]. The deficiency of Hsp60 chaperone in mitochondria is associated with morphological changes, deficient ATP synthesis, and in particular, a defect in the assembly of the respiratory chain complex III in neuronal tissues [46]. Furthermore, the Hsp60 deficiency in human embryonic kidney cells has been shown to inhibit the cell proliferation and decrease the mitochondrial membrane potential [47]. The down-regulation of Hsp60 expression in HEK-293 cells by RNA interference also impaired the biogenesis of medium-chain acyl-CoA dehydrogenase, which was a mitochondrial enzyme involved in the fatty acid metabolism [48]. In the present study, the expression of HSP60 was decreased in islets of diabetic mice and suppressed by low-concentration AGEs exposure in cultured β-cells. Therefore, we assumed that the AGEs-induced mitochondrial dysfunction resulted from insufficient expression of mitochondrial HSP60. We further found that the overexpression of HSP60 could significantly reverse the AGE-induced β-cell hypertrophy and abnormalities of ATP production and insulin secretion. These findings suggest that the mitochondrial HSP60 may be a crucial target protein for AGEs-induced alterations in β-cell morphology and function. Indeed, the present results provided indirect evidence to show that mitochondrial dysfunction and ATP production impairment may contribute to AGEs-induced β-cell hypertrophy. The mechanism for connection between hypertrophy and mitochondrial dysfunction in β-cells during diabetogenic states needs to be further clarified in the future.

The serum levels of glyceraldehyde-derived AGEs, one of the AGEs types, have been suggested to be a biomarker for insulin resistance and diabetic vascular injury [49]. The AGEs-RAGE axis triggers the oxidative stress and results in inducing the inflammatory and thrombogenic reactions that are involved in diabetic vascular complications. Nakamura et al. have also shown that glyceraldehyde-derived AGEs levels are positively associated with soluble form of RAGE (sRAGE) in type 2 diabetic patients, suggesting that the levels of sRAGE may be increased in response to circulating AGEs [50]. In the present study, we also found that low-concentration glyceraldehyde-derived AGEs significantly induced oxidative stress, increased p27^Kip1^ expression and cell hypertrophy, and decreased HSP60 expression, insulin secretion, and ATP content in cultured β cell line, which could be reversed by RAGE neutralizing antibody.

Accumulating evidence shows that phosphoinositide 3-kinase (PI3K)-Akt/PKB signaling plays an important role in promoting hypertrophy, hyperplasia, and neogenesis [51]. The activation of the Akt1/PKBα signaling has been found to increase islet β cell mass by elevation of size and number [52]. Akt signaling pathway has also been shown to be involved in AGEs-RAGE axis-induced inflammation or apoptosis or autophagy in many cell types [53]. The role of Akt signaling in the AGEs-induced islet β-cell hypertrophy/dysfunction and its relationship with HSP60 down-regulation still remains unclear that are needed to clarify in the future.

In conclusion, in this study, we demonstrate for the first time that AGEs-RAGE axis causes mitochondrial dysfunction in the pancreatic islet β-cells by down-regulating the mitochondrial chaperone HSP60 and leads to β-cell hypertrophy and dysfunction. These findings suggest that HSP60 down-regulation may be a pathological link and therapeutic target for AGEs-induced β-cell morphological and functional changes under diabetic hyperglycemia of early stage of T2D.

## MATERIALS AND METHODS

### Animals

Male 12-week-old *db/db* mice and non-diabetic littermate control *db/m^+^* mice were used in animal experiments. The *db/db* and *db/m^+^* mice were purchased from Jackson Laboratories (Bar Harbor, ME, USA). All animal studies were approved by the ethical review committee of National Taiwan University, College of Medicine, and were carried out in accordance with regulations of Taiwan and NIH guidelines on the care and welfare of laboratory animals. After the mice were sacrificed, the pancreas was isolated and the blood samples were collected.

### Serum biochemical analysis

Serum biochemical parameters such as serum glucose and insulin were determined by a commercially available clinical chemistry analyzer (Roche, Mannheim, Germany). Serum AGEs levels were measured by an AGEs competitive ELISA Kit (OxiSelect™, STA-317, Cell Biolabs, San Diego, CA, USA), which used glyceraldehyde-derived AGE-BSA as a standard.

### Histology and immunohistochemistry

The 4-μm-thick paraffin-embedded pancreas tissue section slides were stained with hematoxylin and eosin. For immunohistochemistry, the primary antibodies for p27^Kip1^, AGEs (ab23722; Immunogen: AGE-BSA and AGE-human serum albumin (HSA); Cross-reacts with BSA and HSA alone < 1%; Abcam, Cambridge, MA, USA), HSP60, RAGE, and insulin (Santa Cruz Biotechnology, Santa Cruz, CA, USA) were used. In some experiments, commercial human pancreas tissue slides from normal elderly subject (67 years) and elderly diabetic patient (77 years) were purchased from GeneTex (catalog No.: GTX24611 and GTX21813; Irvine, CA, USA) and stored at room temperature for following immunohistochemical analysis.

### Pancreatic islet isolation

Islets of Langerhans were isolated by collagenase digestion from the mouse pancreas as previously described [54]. After separation on a Ficoll gradient, the islets were further purified by handpicking to eliminate any remaining exocrine tissue. Whole islets were maintained in culture medium consisting of RPMI-1640 medium supplemented with 10% fetal bovine serum and 1% penicillin/streptomycin/amphotericin B at 37°C in an atmosphere of 95% air/5% CO_2_ before experimentation.

### AGEs of bovine serum albumin (AGE-BSA) preparation

AGE-BSA was prepared as previously described [55]. Briefly, BSA (25 mg/ml) was incubated with 0.1 M glyceraldehyde in 0.2 M NaPO_4_ buffer (pH 7.4) for 7 days under sterile conditions. The unincorporated sugars were then removed by PD-10 column chromatography and dialysis against phosphate-buffered saline. Controlling non-glycated BSA was incubated in the same conditions except for the absence of reducing sugars. Identification of AGEs was determined by an Ultraflex-III-MALDI-TOF/TOF mass spectrometry (Bruker Co., Germany). Prepared samples were tested for endotoxin using Endospecy ES-20S system (Seikagaku Co., Tokyo, Japan).

### Cell cultures

Rat pancreatic β-cell line (RINm5f) was purchased from the American Type Culture Collection. Cells passage conditions were followed as manufacturer's procedures. Briefly, The RINm5f cells were maintained in RPMI-1640 supplemented with 10% (v/v) fetal bovine serum (FBS), sodium pyruvate, HEPES, 1.5 g/L sodium bicarbonate, 2 mM L-glutamine, 100 μg/ml streptomycin, and 100 IU/ml penicillin. Culture reagents and mediums were acquired from Gibco (Carlsbad, CA, USA). These cells were incubated at 37°C and 5% CO_2_.

### Cell viability assay

Cell viability was determined by water-soluble tetrazolium-8 (WST-8) assay (Sigma-Aldrich, Louis, MO, USA). Cells (2 × 10^4^) were seeded in 96 well plates at 37^°^C and 5% CO_2_ overnight. Subsequently, the cells were treated with or without AGE-BSA or non-glycated BSA (5-100 μg/ml) for 24 hours, and then 10 μl of WST-8 solution was added to each well. After 3 hours incubation, the absorbance was measured at 450 nm and 650 nm using a microplate reader (Bio-Rad, Hercules, CA, USA).

### Cell diameter measurement

Cell diameter measurements were performed with the Sysmex flow particle image analyzer FPIA-3000 (Malvern Instruments; Worcestershire, UK). Briefly, AGE-BSA-treated cells were harvested by trypsin digestion and suspended in 1 ml phosphate-buffered saline (PBS) for injecting in a sample chamber and homogenized by a mixing rotor. The cells were then dispersed in an electrolytic sheath solution and were guided to a transparent flow cell where they were irradiated by pulsed light to take images of particles. The cells were captured with a *charge coupled device* (CDD) camera and, by image analysis using the FPIA-3000 software; information on the number, the size and shape of the particles was obtained. The size of cell was evaluated by the equivalent circle diameter (EC diameter) which is the diameter of the circle having the same projected area (*S*) as the particle image (EC diameter = 2 × (*S*/*π*)^1/2^). Good accuracy in diameter measurements can be obtained by this technique; with standard particles of 10 ml (latex microspheres from Duke Scientific Corporation), measured 10 times in succession, a standard variation of 0.5% was obtained for the average diameter.

### Trypan blue cell counting

Cells (2 × 10^5^) were seeded in 6-well plates at 37°C and 5% CO_2_ overnight. Subsequently, the cells were treated with or without AGE-BSA or non-glycated BSA (0.1-1 μg/ml) for 24 hours, and then cells were trypsinized and washed twice with PBS. Cells were stained with trypan blue. Viable (unstained) and nonviable (blue stained) cells were counted with an automated cell counter (Invitrogen, Carlsbad, CA, USA).

### Cellular hypertrophy analysis

Equal numbers of cells were lysed and measured the total protein content using the Bio-Rad protein assay kit. Total protein was expressed as micrograms of protein per 10^4^ cells.

### Area of cell and islet counting assay

For counting the RINm5f cells area and the areas of islets and β-cells in islets of diabetic patient and *db/db* mice, the RINm5f cells and islets in stained pancreatic slides were analyzed using a blinded fashion with a digital image analysis software (ImageJ version 1.48, National Institutes of Health, USA) [56].

### Immunoblotting

The detection of protein expressions in cells and tissues were performed by Western blotting as described previously. Cells and tissues were lysed in the buffer containing 150 mM NaCl, 1% NP-40, 0.1% SDS, 50 mM Tris-HCl (pH 8.0). The protein samples were separated by SDS-PAGE and transferred onto the Immobilon P membranes (Millipore Technology, Billerica, MA, USA). After blocking with for 5% skin milk solution for 2 hours, the membranes were incubated overnight at 4^°^C with primary antibodies for p27^Kip1^ (1:1000; Santa Cruz Biotechnology, Inc., SC, CA, USA), AGEs (1:2000; ab23722, Abcam, Cambridge, MA, USA), HSP60 (1:3000), RAGE (1:2000), and lamin A (1:3000) (Sigma-Aldrich). Follow, the secondary antibody were incubated for 1 hour and the membranes were detected by using enhanced chemiluminescence (ThermoFisher, Con, CO, USA) on LAS-4000mini performing system (Fuji Film, Tokyo, Japan). The blotting bands were quantified by densitometric analysis using Multi Gauge v3.2 software (Fuji Film, Tokyo, Japan).

### DNA transfection

The pM51-HSP60 plasmids were provided from GeneCopoeia (Rockville, MD, USA). The full-length cDNA of *HSP60* (Gene ID: 63868) was inserted into the XmnI-NotI sites of the pReceiver-M51 (pM51) vector, and the inserted fragment was confirmed by sequencing. Cells were plated in 60 mm plates 24 hours prior to transfection. Cells were then transfected with 1-4 μg of the indicated plasmids using PolyJet™ DNA *In Vitro* Transfection Reagent (SignaGen Laboratories, Rockville, MD, USA) according to the manufacturer's instructions. After 8 hours transfection, the transfection medium was removed and complete medium was added to recover cell growth for 48 hours. After further treatment, the cells were harvested and western blot assay, cell viability and cell size detection were performed.

### ATP production

The ATP production measurement was performed by a luminescence ATP detection assay system (PerkinElmer, Waltham, MA, USA). Cells were cultured in 96 well plates at 37^°^C and 5% CO_2_. After 24 hours pre-incubation, each well which containing 1×10^4^ cells were treated with 30 μg/ml AGE-BSA or non-glycated BSA for 24 hours. Cells or the pancreatic islets of *db/db* and *db/m^+^* were then lysed in the buffer containing 150 mM NaCl, 1% NP-40, 0.1% SDS, 50 mM Tris-HCl (pH 8.0) and used for ATP levels measurement according to the manufacturer's instructions. Results were normalized to protein content. Each experimental data point represents the mean of duplicate wells from three independent experiments.

### Insulin content and secretion

Cells were washed two times with Krebs-Ringer bicarbonate buffer (KRBB, 129 mM NaCl, 4.8 mM KCl, 1.2 mM MgSO_4_, 1.2 mM KH_2_PO_4_, 2.5 mM CaCl_2_, 5 mM NaHCO_3_, 0.1% BSA, 10 mM HEPES, (pH 7.4) and 2.8 mM glucose) and incubated in KRBB for 1 hour. For insulin secretion assay, the supernatant fraction was collected for insulin values detection. For insulin content assay, the cells were lysed for insulin values measurement. The insulin values were detected by a High Range Rat Insulin ELISA Kit (DRG Instruments GmbH, Marburg, Germany) and normalized to protein content as determined by BCA assay (Pierce, Rockford, IL, USA).

### Measurement of reactive oxygen species (ROS)

The ROS measurement was performed by a Hydrogen Peroxide Assay Kit (BioVision, Milpitas, CA, USA). Cells were treated with or without AGE-BSA or non-glycated BSA (0.5-50 μg/ml) for 24 hours in the presence or absence of RAGE neutralizing antibody (20 μg/ml). In some experiments, the pancreatic islets of *db/db* and *db/m^+^* mice were isolated and lysed in in the buffer containing 150 mM NaCl, 1% NP-40, 0.1% SDS, 50 mM Tris-HCl (pH 8.0). Measurements were performed according to the manufacturer's protocol and quality control was ensured.

### Statistical analysis

Data are expressed as means ± SEM. The significant difference from the respective controls for each experimental test condition was assessed by one-way analysis of variance (ANOVA) and Dunnett test. The difference is significant if the *P*-value is less than 0.05. Statistical analysis was performed using GraphPad Prism V5.01 software (GraphPad Software Inc., La Jolla, CA, USA).
